# On Critical States, Rupture States and Interlocking Strength of Granular Materials

**DOI:** 10.3390/ma10080865

**Published:** 2017-07-27

**Authors:** Chris M. Szalwinski

**Affiliations:** 302-100 Quebec Avenue, Toronto, ON M6P4B8, Canada; chris.szalwinski@gmail.com; Tel.: +1-416-763-4020

**Keywords:** internal friction, cohesion, soil mechanics, bulk solids’ flow, over-compaction, interlocking, jammed state, rupture, slip

## Abstract

The Mohr-Coulomb theory of strength identifies cohesion and internal friction as the two principal contributions to the shear strength of a granular material. The contribution of cohesion in over-compacted granular materials has been challenged and replacing cohesion with interlocking has been proposed. A theory of rupture strength that includes interlocking is derived herein. The physics-chemistry concept of critical state is elaborated to accommodate granular materials, based on empirical definitions established in the fields of soil mechanics and bulk solids’ flow. A surface in state space, called the critical compaction surface, separates over-compacted states from lightly compacted states. The intersection of this surface with the Mohr-Coulomb envelope forms the critical state surface for a granular material. The rupture strength of an over-compacted granular material is expressed as the sum of cohesion, internal friction and interlocking strength. Interlocking strength is the shear strength contribution due to over-compaction and vanishes at critical state. The theory allows migrations from one critical state to another. Changes in specific volume during such migrations are related to changes in mean-normal effective stress and uncoupled from changes in shearing strain. The theory is reviewed with respect to two established research programs and underlying assumptions are identified.

## 1. Introduction

The particulate nature of granular materials distinguishes them from ideal solids and fluids and introduces an order of complexity to mechanical descriptions that is absent in the descriptions for solids and fluids. Finite element software incorporates the mechanical properties of material bodies through so-called constitutive relations. The modern version of this analytical software can predict three-dimensional stress distributions across continuous representations of those bodies. For bodies that include some over-compacted material, these relations require expressions for rupture strength, as the material dilates; critical strength, as it flows; and functions that describe variations in each of these strengths as shearing mode changes.

This paper develops a theory of the rupture strength of an over-compacted granular material and addresses several issues related to the Mohr-Coulomb theory of strength that researchers have raised over the last three decades. The Mohr-Coulomb theory of strength has been the most widely used theory of strength amongst engineers [1]. In 1773, Charles Augustin de Coulomb defined the shear strength of a material as the sum of cohesion and internal friction on a slip plane [2]. Cohesion is the contribution to shear strength related to the area of the plane, and is independent of the normal force on it. Internal friction is the contribution related to the normal force on the plane, and is independent of its area. Jacques Heyman has noted that it was precisely the introduction of two parameters describing the soil properties that is of the utmost importance in Coulomb’s analysis and that no writer before Coulomb seems to have introduced the possible cohesion of the soil into his theory [3].

In 1900, Otto Mohr expressed limiting states of failure in the form of an envelope on the set of admissible stress states [4]. In 1943, Karl Terzaghi applied this theory to over-compacted soils. He reported values of cohesion and internal friction for those soils that differ from the values for normally compacted soils. He named the over-compacted values the ‘true’ properties of a soil and assumed perfect plasticity at failure [5].

Over-compacted granular materials exhibit a feature that is not readily apparent in solid or fluid materials. In 1885, Osborne Reynolds showed that dense sands expand when sheared before failure and introduced the term *dilatancy* to describe this effect [6]. In 1948, Donald Taylor reported the results of studies on volume change during continuous shearing of dense sands to failure. He called the ratio of volume change to shearing strain *interlocking* [7]. Ten years later, Kenneth Roscoe, Andrew Schofield and Peter Wroth reported the results of shearing remolded, reconstituted soils from different initial densities [8]. They identified the final states at which densities and stress attain constant values as the critical states of their soils. The following year, Andrew Jenike and Richard Shield proposed constitutive relations that predict reductions in the shear strength of a bulk solid as it dilates, and that identify its shear strength once it has reached a steady flow state [9]. In 1962, Peter Rowe reported the results of studies on regularly packed assemblies of particles and distinguished the discrete planes of inter-particle sliding from an imaginary plane of interlocking [10]. He assumed that the orientations of the discrete planes align with the plane of interlocking at ultimate failure. In 1968, Schofield and Wroth reported the results of normalizing data for the rupture strength of over-compacted Weald clay with respect to the mean-normal effective stress at critical state [11,12]. This revealed the linear dependency of rupture strength on critical mean-normal effective stress.

Roscoe and Jenike discussed their respective research programs at Cambridge in 1968 and agreed that they were modeling the same class of material [13]. Each had developed a distinct solution within the framework of the mathematical theory of plasticity [14]. Plasticity theory is commonly used to frame constitutive relations in finite element software. The theory represents a specimen of the material as a homogeneous element, the strain of which is spatially constant at all length scales. Modeling a granular material as a homogeneous element requires special attention to features that distinguish it from ideal solids. Ian Collins, Balasingam Muhunthan, and B. Qu have noted that one such fundamental feature is the absence of a unique set of unloaded reference states [15]. They also noted that, although seldom explicitly stated, an implicit assumption in all critical-state models is the adoption of a critical-state as the reference state.

In 2006, Schofield rejected Terzaghi’s identification of the strength of over-compacted soil as the sum of his ‘true’ cohesion and ‘true’ internal friction. He proposed defining the peak strength of a remolded, re-consolidated fine-grained soil as the sum of its ultimate critical-states, drained friction and Taylor’s interlocking [16,17]. Richard Dean, in his reply, requested a broader definition that applies to the undisturbed or partially disturbed soils typically encountered in engineering [18].

The Cambridge and Jenike research programs defined the locus of critical states using similar, but not identical, equations. In the Cambridge program, a critical state is a state on a line described by the intersection of a critical-compaction surface defined in semi-logarithmic space and the cohesionless version of the Drucker-Prager yield surface [19]. No volume or effective stress changes occur at critical state. In the Jenike program, a critical state is a state on a surface described by the intersection of a critical-compaction surface in logarithmic-logarithmic space and the Mohr-Coulomb yield surface. Contractions occur as stresses decrease in steady convergent flow; expansions occur as stresses increase in steady divergent flow [20]. In 2009, Zhen-Yu Yin and Ching Chang tabulated data for a variety of clays, showing that, for some clays, the critical-compaction surface occupies different positions for triaxial compression and triaxial extension [21]. These data clearly show that the locus of critical states may be a function of the shearing mode. In other words, the locus of critical states describes a surface rather than a line in state space.

Researchers have studied the mechanics of dilation in granular materials for some time [22,23,24,25]. In 2007, Collins and his collaborators [26] showed that if Reynolds’ dilatancy is represented as an internal kinematic constraint, the resulting model is anisotropic, and the concept of a critical state must be replaced by the more general notion of a Reynolds-Taylor state. In 2010, Collins and his collaborators [15] introduced a state-based ratio to measure the fraction of applied plastic work being released during dilation, noting that other researchers had proposed different parameters to measure dilation [27,28].

This paper proposes a theory of rupture strength that unifies these theories. The development introduces the normal compaction strength as the independent state parameter, and distinguishes the orientation of the plane of rupture from the plane of slip. The first sub-section below bases its definition of a critical state on the accepted multi-phase definition for solids and fluids used in physics and chemistry. The definition proposed here merges two conditions: (a) a condition that relates the mean-normal effective stress, the shearing mode and the normal compaction strength at a multi-phase state; and (b) a condition that relates the shear strength to the mean-normal effective stress at a multi-phase state. States that satisfy both conditions lie on the critical-state surface for the material. The second sub-section derives interlocking strength at rupture as the product of over-compaction and a material property that describes the difference in orientations of the planes of rupture and slip. Interlocking strength vanishes at critical state. Rupture strength is the sum of cohesion along the slip plane, internal friction along the slip plane, and this interlocking strength. The discussion section reviews the theory developed here with respect to the predecessors listed above and compares them to one another.

## 2. Results

### 2.1. Critical-Compaction Surface

In the field of condensed matter physics, a critical state is a state of a system at which physical differences between two phases vanish [29]. The necessary condition for a critical state is the identity of the state descriptors for the two phases. Here, those phases are the solid and fluid phases of a granular material.

Let us represent a body of granular material by a continuum model and a sample of the material within this body by an infinitesimal element of this continuum model. The mean-normal effective stress on the element is the average of the major (σ_1_’), intermediate (σ_2_’) and minor (σ_3_’) principal effective stresses:

p’ ≡ (σ_1_’ + σ_2_’ + σ_3_’)/3. (1)

Effective stress is the difference between Cauchy stress and the fluid pressure within the interstices of the sample. Positive-valued normal stress is compressive. The shearing mode is defined as

ω ≡ (σ_2_’ − σ_3_’)/(σ_1_’ − σ_3_’). (2)

In the absence of shear stress, the shearing mode is indeterminate.

Let us describe the arrangement of the particles within the sample in terms of the limiting ratio of its volume to the volume occupied by the particles alone, as that volume vanishes. This ratio is called the representative element’s specific volume:

(3) $$\nu \equiv \underset{\Delta V_{p\;\to \;0}}{\lim}\Delta V/\Delta V_{p},$$

where ΔV is the volume of the element and ΔV_p_ is the volume of the particles within the sample.

**Figure 1:**
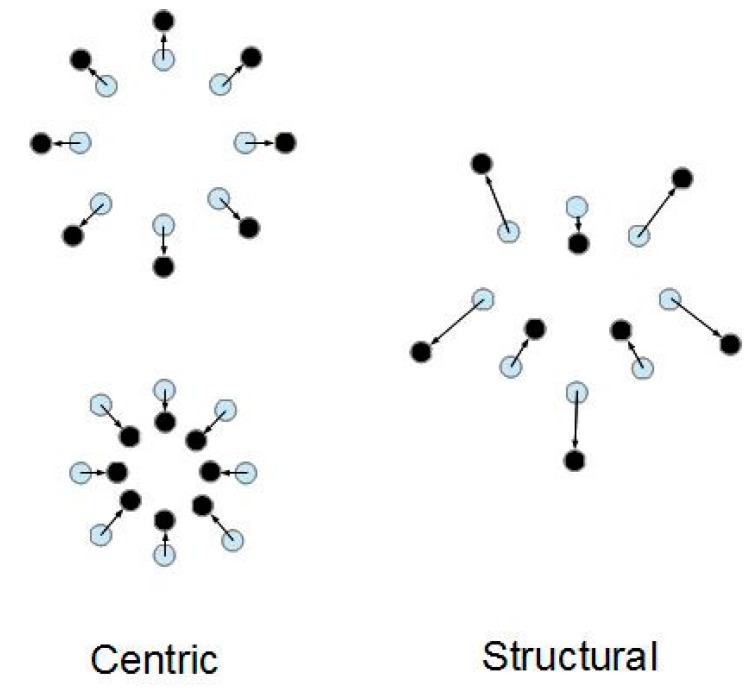
Centric and structural components of specific volume change.

While ideal solids and fluids exhibit unique densities in unstressed states, granular materials exhibit no such reference states. To compensate for this missing feature, let us introduce a relation between the specific volume of the element and changes in the mean-normal effective stress:

dν + f(p’, ν)·dp’ = 0,(4)

where f(p’, ν) is the apparent flexibility, which is empirically determined. Changes in specific volume can be distinguished into two categories: certain rearrangements preserve the inter-particle contact structure, while others mutate that structure (see Figure 1). Accordingly, let us describe a general change in the specific volume of the element as a change consisting of two components:

dν = dν_c_ + dν_s_. (5)

The centric component dν_c_ describes the uniform radial deformation that preserves the inter-particle contact structure. The structural component dν_s_ describes its mutations.

**Figure 2:**
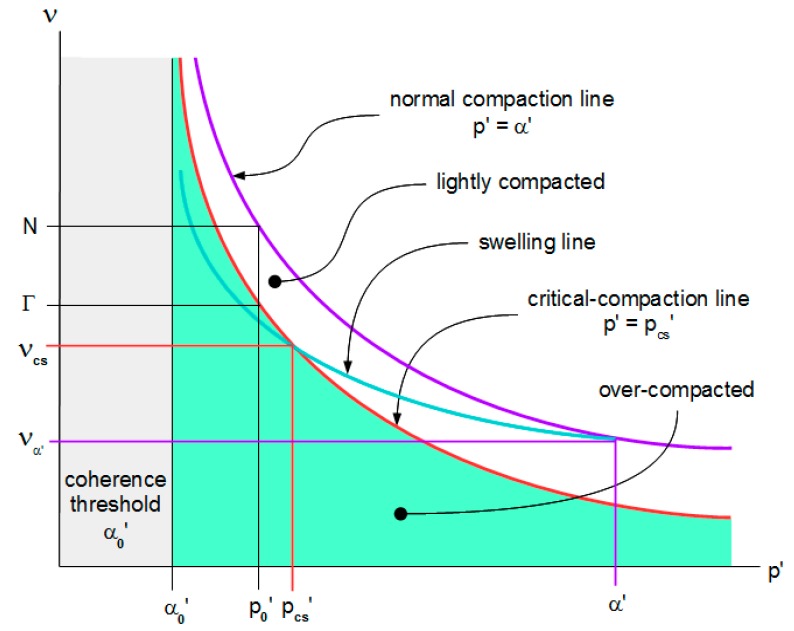
Normal compaction, swelling and critical-compaction lines.

The centric component is reversible and directly related to the change in mean-normal effective stress:

dν_c_ = −f_c_(p’, ν)·dp’, (6)

where f_c_(p’, ν) is the centric flexibility. It is empirically determined as the tangential slope of the swelling line in ν-p’ space (see Figure 2). The structural component is the non-centric part of the specific volume change. This component is irreversible, dissipates energy, and follows directly from Equations (4)–(6):

dν_s_ = −[f(p’, ν) − f_c_(p’, ν)]·dp’. (7)

Each component of specific volume change can be negative-valued as well as positive-valued.

Changes to the inter-particle contact structure of the sample alter its strength. Let α’ denote the maximum mean-normal effective stress that the element supports in the absence of any shear stress. Let us call α’ the element’s normal compaction strength, or more briefly, its normal strength. The normal compaction line in ν-p’ space relates this normal strength to the element’s specific volume. This line’s tangential slope is the apparent flexibility f(p’, ν) in Equation (4). The change in the normal strength of the element is directly related to the structural component of the change in its specific volume:

dα’ = −dν_s_/[f(p’, ν) − f_c_(p’, ν)]. (8)

Let us call this relation the *transmutation* relation for the element.

The relation between the element’s specific volume and the mean-normal effective stress is not unique, but depends on the element’s normal strength. To formulate this relation, let us select a reference state (Ν,p_0_’) on the normal compaction line, where Ν and p_0_’ are that state’s specific volume and mean-normal effective stress respectively (see Figure 2). A process from this reference state to another normal compaction state (ν_α’_,α’) alters the element’s normal strength. The change in specific volume follows from Equation (4):

(9) $$\int_{\nu_{\alpha^{\prime }}}^{N}d\nu =-\int_{p_{0^{\prime }}}^{\alpha^{\prime }}f\left(p^{\prime },\nu \right)\cdot {\mathrm{dp}}^{\prime }.$$

In a subsequent centric process from state (ν_α’_,α’) to state (ν,p’), the distance between adjacent particles increases but the inter-particle contact structure remains unchanged. Hence, the element’s strength remains unchanged. The integral of Equation (6) with Equation (5) gives

(10) $$\int_{\nu_{\alpha^{\prime }}}^{\nu }d\nu =-\int_{\alpha^{\prime }}^{p^{\prime }}f_{c}\left(p^{\prime },\nu \right)\cdot {\mathrm{dp}}^{\prime }.$$

The net change in specific volume from reference state (Ν,p_0_’) is the sum of these two processes:

(11) $$\int_{\nu }^{N}d\nu =-\int_{p_{0^{\prime }}}^{\alpha^{\prime }}f\left(p^{\prime },\nu \right)\cdot {\mathrm{dp}}^{\prime }-\int_{\alpha^{\prime }}^{p^{\prime }}f_{c}\left(p^{\prime },\nu \right)\cdot {\mathrm{dp}}^{\prime }.$$

The first term on the right-hand side captures all of the structural changes for the process set, while the second term corrects (reduces or augments) the centric changes of the first term. Equation (11) holds for any state in the solid phase.

The relation between the element’s specific volume and the mean normal effective stress during continuous flow may vary with the shearing mode [21]. To formulate this relation, let us select a reference state (Γ(ω),p_0_’) on the locus of continuous-flow states, where Γ(ω) is the specific volume for shearing mode ω at the same reference mean-normal effective stress p_0_’ as selected for the solid phase processes. Let us call this locus the critical-compaction locus (see Figure 2). A process from this locus’ reference state to another flow state under the same shearing mode ω alters the element’s specific volume. The change in specific volume follows from Equation (4):

(12) $$\int_{\nu }^{\Gamma \left(\omega \right)}d\nu =-\int_{p_{0^{\prime }}}^{p^{\prime }}f\left(p^{\prime },\nu \right)\cdot {\mathrm{dp}}^{\prime }.$$

The reference specific volume for any state in the fluid phase is distinct from the reference specific volume for any state in the solid phase.

At a critical state, the material in its solid and fluid phases supports the same specific volume and mean-normal effective stress. The states that satisfy this particular condition are the critical-compaction states for shearing mode ω. Subtracting Equation (11) from Equation (12) yields

(13) $$\int_{N}^{\Gamma \left(\omega \right)}d\nu =-\int_{p_{{\mathrm{cs}}^{\prime }}}^{\alpha^{\prime }}\;\left[f\left(p^{\prime },\nu \right)-f_{c}\left(p^{\prime },\nu \right)\right]\cdot {\mathrm{dp}}^{\prime },$$

where p_cs_’ is the mean-normal effective stress at critical state, or, more briefly, the critical-compaction strength.

At sufficiently low mean-normal effective stress, the inter-particle contact structure loses its coherence. Let α_0_’ denote this coherence threshold, below which the flexibilities are no longer well-defined. For a material with linear flexibilities in ν-ln p’ space:

f(p’, ν) = λ/(p’ − α_0_’)  α_0_’ < p’ ≤ α’, (14)

f_c_(p’, ν) = κ/(p’ − α_0_’)  α_0_’ < p’ ≤ α’, (15)

where λ and κ are, respectively, the tangential slopes of the normal compaction and swelling lines in ν-ln p’ space. Substituting these expressions into Equation (13) gives the element’s critical-compaction strength:

p_cs_’ = a(ω)·(α’ − α_0_’) + α_0_’ = a(ω)·α’ + [1 − a(ω)]·α_0_’, (16)

where a(ω) is the material’s phase-transition function

a(ω) = e^−[N−Γ(ω)]/(λ−κ)^, (17)

and e denotes the exponential function. For a material with linear flexibilities in ln ν-ln p’ space:

f(p’, ν) = β·ν/(p’ − α_0_’)  α_0_’ < p’ ≤ α’, (18)

f_c_(p’, ν) = ζ·ν/(p’ − α_0_’)   α_0_’ < p’ ≤ α’. (19)

where β and ζ are, respectively, the tangential slopes of the normal compaction and swelling lines in ln ν-ln p’ space. Substituting into Equation (13) gives Equation (16) where

a(ω) = [Γ(ω)/N]^−^^1/(β−ζ^^)^. (20)

The phase-transition function a(ω) relates the critical-compaction strength of the material to its normal compaction strength.

The critical-compaction surface defined by Equation (16) partitions p’-α’-ω space into two non-overlapping domains. Normally and lightly compacted conditions satisfy

p_cs_’ < p’ ≤ α’. (21)

Over-compacted conditions satisfy

α_0_’ < p’ < p_cs_’. (22)

**Figure 3:**
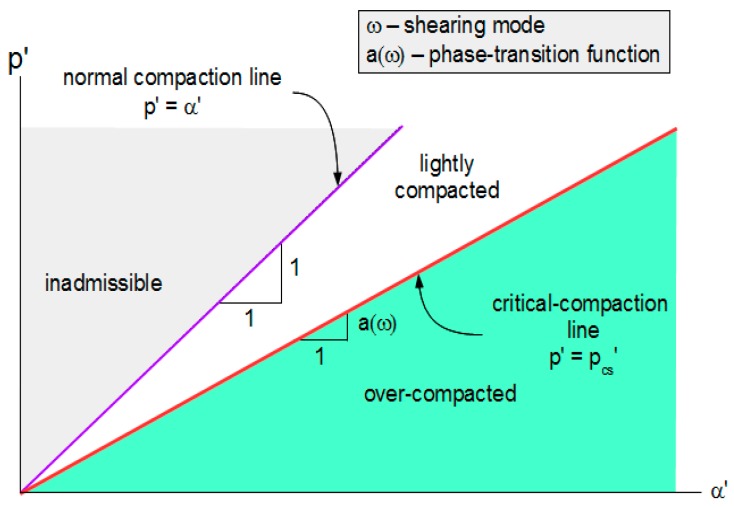
Partitioning of p’-α’-ω space into lightly compacted and over-compacted domains.

Figure 3 illustrates this partitioning of domains.

### 2.2. Critical-State Surface

Unlike ideal fluids, granular materials sustain shear stress in extremely slow flow conditions. Equation (16) leaves shear capacity undefined. Taken alone, this critical-compaction relation does not identify the locus of critical states uniquely. To complete the definition of a critical state for a granular material, we also require its mobilization condition.

The Mohr-Coulomb theory of strength provides a simple form of this condition. It assumes that the intermediate principal effective stress does not contribute to the element’s shear strength. The center and radius of Mohr’s stress circle are defined by

σ’ ≡ (σ_1_’ + σ_3_’)/2, (23)

r ≡ (σ_1_’ − σ_3_’)/2, (24)

where r is the maximum shear stress on the element.

**Figure 4:**
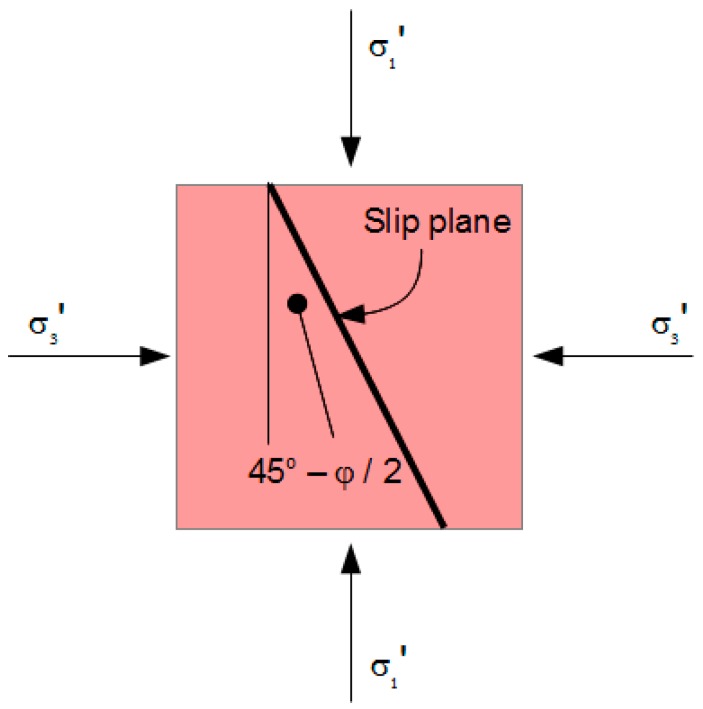
The Slip Plane.

The shear stress that mobilizes internal friction in a flowing Mohr-Coulomb material is the sum of cohesion c_s_ and internal friction on the slip plane:

τ_s_ = c_s_ + σ_ns_’·tan φ, (25)

where τ_s_ is the shear strength along the slip plane, σ_ns_’ is the normal effective stress component on the slip plane and φ is the angle of internal friction of the slip plane (see Figure 4). Any stress circle that touches mobilization Equation (25) has radius

r = (σ’ + c_s_·cot φ)·sin φ. (26)

The shear strength expressed in terms of σ’ is given by

τ_s_ = (c_s_ + σ’·tan φ)·cos^2^ φ. (27)

This is an equivalent expression for the required mobilization condition.

The center of any stress circle is related to the mean-normal effective stress:

σ’ = p’ − r·(2ω − 1)/3.(28)

Substituting Equation (26) into Equation (28) gives σ’ in terms of p’ and ω:

σ’ = g(ω)·[p’ − c_s_·cos φ·(2ω − 1)/3], (29)

where g(ω) is the intermediate principal effective stress factor

g(ω) ≡ 1/[1 + sin φ·(2ω − 1)/3].(30)

Note that, for an intermediate principal effective stress that is equal to the average of the major and minor principal effective stresses (ω = 1/2), σ’ is the mean-normal effective stress and g(ω) is unit-valued. Assuming this shearing mode in an exposition simplifies that exposition without hiding any underlying concept.

Substituting Equation (16) into Equation (29) expresses σ’ at critical state in terms of normal strength:

σ_cs_’ = g(ω)·{a(ω·α’ + [1 − a(ω)·α_0_’ − c_s_·cos φ·(2ω − 1)/3}. (31)

**Figure 5:**
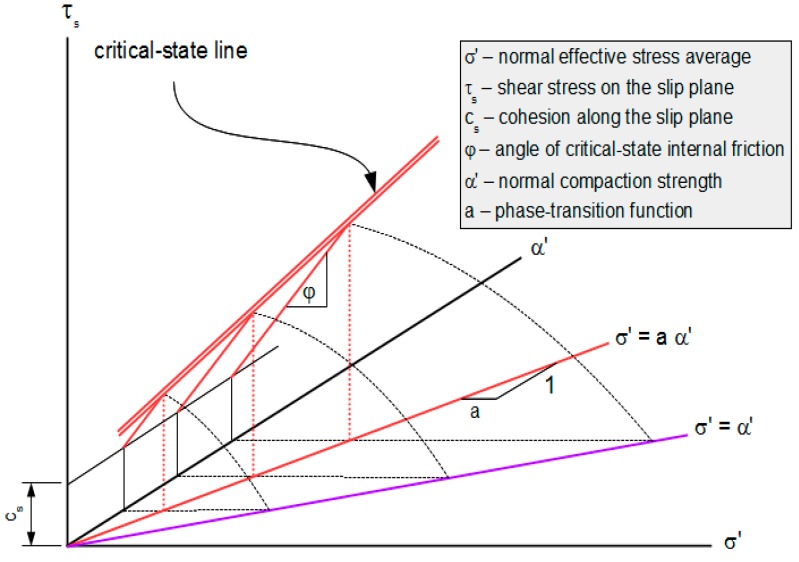
Critical-state line for shearing mode ω = 1/2.

This is the critical-compaction relation for a Mohr-Coulomb material. The surface defined by this relation is its critical-compaction surface. The intersection of this surface and the mobilization envelope defined by Equation (27) is the material’s critical-state surface. The critical-state line on that surface corresponding to ω = 1/2 is illustrated in Figure 5.

At any critical state, any change in the element’s specific volume due to a change in stress that keeps the element’s state on the critical-state surface follows from Equation (4) and the derivatives of Equations (29) and (30):

dν_cs_ = − f(p’, ν)·[dσ_cs_’/g(ω) + (2/3) (c_s_·cos φ + σ_cs_’·sin φ)·dω_cs_]. (32)

Shearing at constant mode supports changes in specific volume that are directly related to changes in the mean-normal effective stress

dν_cs_ = − f(p’, ν)·dσ_cs_’/g(ω)   for dω = 0. (33)

### 2.3. Rupture Surface

Schofield and Wroth noted the absence of data in both high and low stress-ratio domains [11,30]. Rupture states lie within the subrange

p_f_’ ≤ p’ ≤ p_cs_’, (34)

where p_f_’ denotes the mean-normal effective stress at fracture. Fracture states lie within the subrange

α_0_’ ≤ p’ ≤ p_f_’. (35)

The expression for p_f_’ is derived below.

To model the dependency of rupture strength on normal compaction strength [11,12], let us augment the Mohr-Coulomb relation with a linear normal compaction term. Then, the material fails on the rupture plane when the shear stress reaches

**Figure 6:**
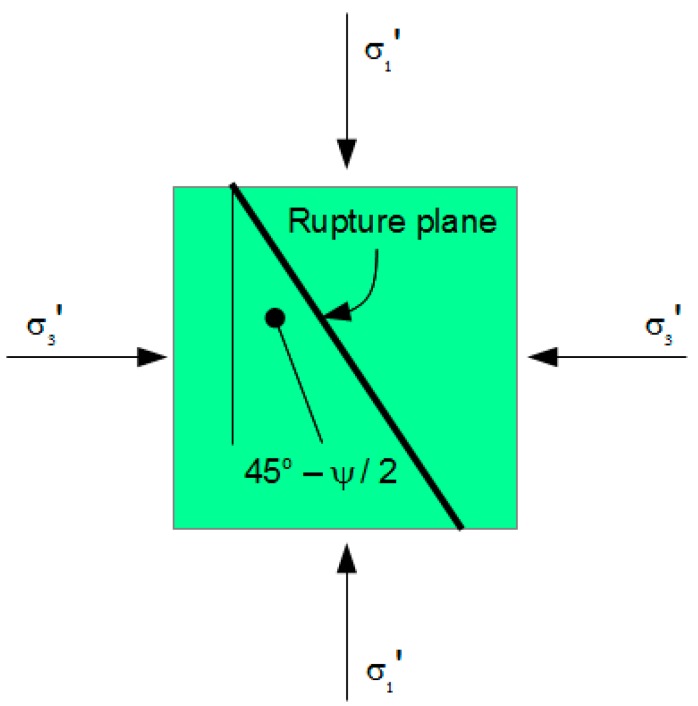
The Rupture Plane.

τ_r_ = c_r_ + σ_nr_’·tan ψ + m·(α’ − α_0_’)  for p_f_’ ≤ p’ ≤ p_cs_’, (36)

where τ_r_ is the shear strength of the rupture plane, c_r_ is the virtual cohesion along the rupture plane, σ_nr_’ is the normal effective stress component on the plane, ψ is the angle of internal friction of the plane and m is the coefficient of compaction influence. The rupture plane is oriented at angle 45-ψ/2 to the major principal axis (see Figure 6). Any stress circle that touches Envelope (36) has radius

r = [σ’ + c_r_·cot ψ + m·(α’ − α_0_’) cot ψ]·sin ψ. (37)

The shear strength expressed in terms of σ’ is given by

τ_r_ = [c_r_ + σ’·tan ψ + m·(α’ − α_0_’)]·cos^2^ ψ. (38)

This is an equivalent condition for mobilization of internal friction along the rupture plane.

**Figure 7:**
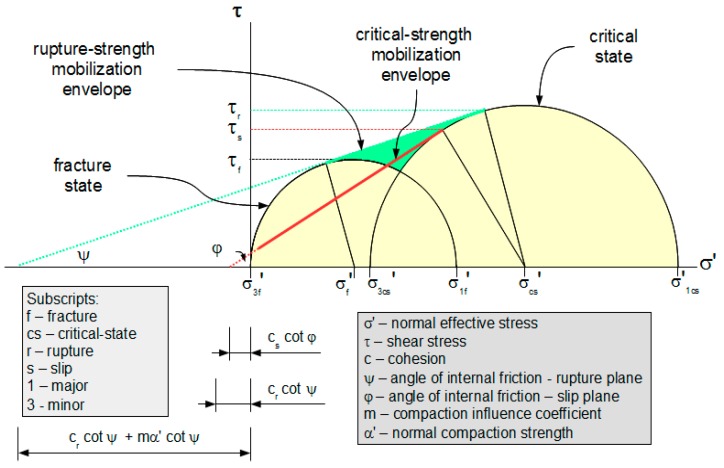
Rupture strength and critical strength at critical state.

At critical state, the material can either rupture or slip and the shear strengths of the rupture and slip planes are related (see Figure 7)

τ_r_ = τ_s_·cos ψ/cos φ  for σ’ = σ_cs_’. (39)

Substituting Equations (27), (31) and (38) into Equation (39) yields

c_s_·cos φ[1 − g(ω)·χ·cos ψ·(2ω − 1)/3] = c_r_·cos ψ + [m − a(ω)·g(ω)·χ]cos ψ·α’ − [m − a(ω)·g(ω)·χ]cos ψ·α_0_’ − g(ω)·χ·cos ψ·α_0_’, (40)

where χ is a material constant given by

χ ≡ (sin φ − sin ψ)/cos ψ.(41)

Since the cohesions and internal-friction angles are independent of the normal strength, the brackets-enclosed part of the second term on the right-hand side of Equation (40) must vanish. Therefore, the coefficient of compaction influence is given by

m = a(ω)·g(ω)·χ.(42)

Equation (40) simplifies to

c_r_·cos ψ = c_s_·cos φ[1 − g(ω)·χ·cos ψ·(2ω − 1)/3] + g(ω)·χ·cos ψ·α_0_’. (43)

Substituting Equation (42) into Equation (36) gives

τ_r_ = c_r_ + σ_nr_’·tan ψ + χ·a(ω)·g(ω)·(α’ − α_0_’)  for p_f_’ ≤ p’ ≤ p_cs_’. (44)

The first and second terms on the right-hand side are independent of the angle of internal friction on the slip plane (φ). The third term is independent of the cohesion and the normal force on the rupture plane. χ represents the difference in orientations of the rupture and slip planes. 

Substituting Equation (42) into Equation (38) gives

τ_r_ = c_r_·cos^2^ ψ + σ’·sin ψ·cos ψ + χ·a(ω)·g(ω)·(α’ − α_0_’)·cos^2^ ψ for p_f_’ ≤ p’ ≤ p_cs_’. (45)

Rearranging the last two terms on the right-hand side and substituting Equation (43) gives

τ_r_ = [c_s_·cos φ/cos ψ + σ’·tan φ + χ·(σ_cs_’ − σ’)]·cos^2^ ψ   for p_f_’ ≤ p’ ≤ p_cs_’. (46)

The first term within the brackets is the virtual cohesion along the slip plane, the second term is the internal-friction along the slip plane, and the third term is the *interlocking strength*. Accordingly, let us call χ the coefficient of interlocking strength. The factor (cos^2^ ψ) adjusts for the conversion of normal stress from σ_nr_’ to σ’.

Fracture conditions truncate the rupture surface at high stress-ratios. Let us define the fracture surface as the locus of states that exhibit zero normal effective stress on some plane through the element

τ_f_ = σ’ = r  for α_0_’ ≤ p’ ≤ p_f_’, (47)

where τ_f_ is the maximum shear stress at fracture. Substituting Equation (37) into Equation (47) gives σ’ at the intersection of the fracture and rupture surfaces:

σ_f_’ = [c_r_ + m·(α’ − α_0_’)]·cos ψ/(1 − sin ψ). (48)

Substituting Equation (31), (42) and (43) into Equation (48) expresses σ_f_’ in terms of σ_cs_’:

σ_f_’ = (c_s_ + χ·σ_cs_’)·cos ψ/(1 − sin ψ). (49)

Substituting Equation (47) into Equation (25) gives the mean-normal effective stress at any fracture state:

p’ = 2σ’·(1 + ω)/3.(50)

Substituting Equation (49) into Equation (50) gives the mean-normal effective stress at the intersection of the fracture and rupture surfaces:

p_f_’ = (2/3)·(1 + ω)·(c_s_ + χ·σ_cs_’)·cos ψ/(1 − sin ψ). (51)

**Figure 8:**
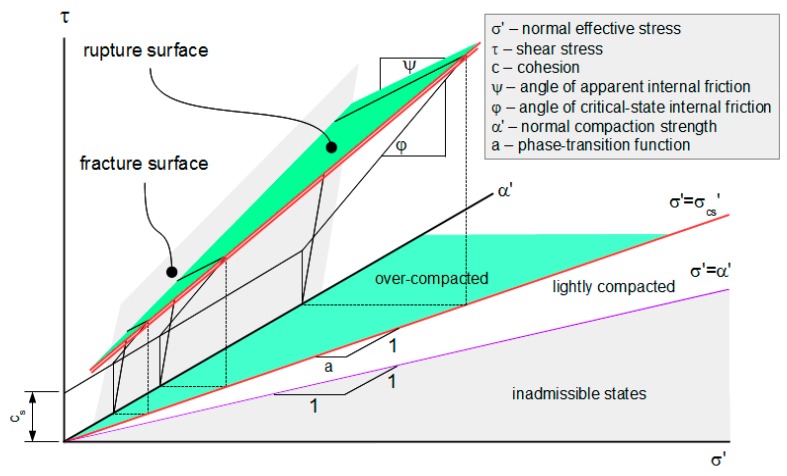
Rupture surface for shearing mode ω = 1/2 and coherence threshold α_0_’ = 0.

**Figure 9:**
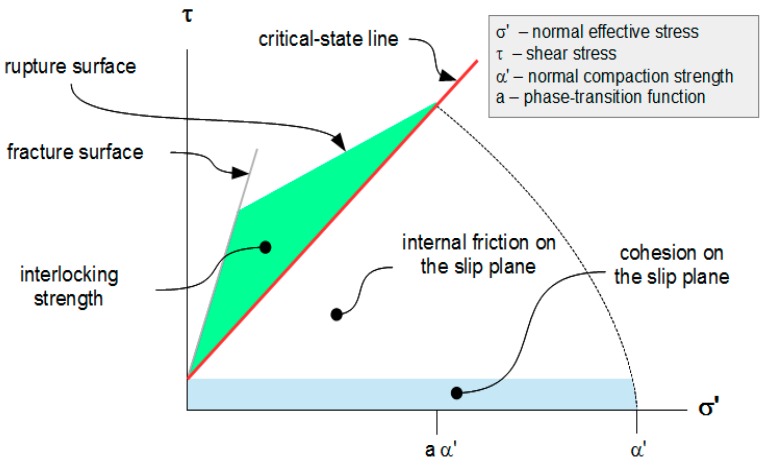
Projection of rupture surface for shearing mode ω = 1/2 and coherence threshold α_0_’ = 0.

The rupture surface for ω = 1/2 and α_0_’ = 0 is shown in Figure 8. Its projection to the σ’-τ plane is shown in Figure 9.

## 3. Discussion

### 3.1. Strength

The present theory is a bounded theory of rupture strength. Its bounding conditions are fracture and critical flow at high and low stress ratios respectively. Similar to the theories of Coulomb, Terzaghi and Jenike, the present theory is independent of kinematics. Its interlocking strength contributes to shear strength in linear proportion to normal compaction. The coefficient of interlocking strength measures the difference in the orientations of the material’s rupture and slip planes. The associated state parameter measures the difference between the critical mean-normal effective stress and the mean-normal effective stress. By the definition of stress at critical state, the shear and normal stress components on the rupture plane are greater than their counterparts on the slip plane. Since the work done in slip is less than the work done in rupture, slip is necessarily more efficient than rupture. Hence, slip is the preferred condition at critical state.

The present theory defines the critical state of a material sample as the multi-phase state that corresponds to the sample’s normal compaction strength. Selecting normal compaction strength as the reference state for the material avoids a teleological reference and enables description of critical state as a function of shearing mode. The necessary and sufficient conditions for a critical state are critical compaction and mobilization of internal friction. The critical compaction condition depends on the normal compaction strength of the material and the applied shearing mode.

The proposed definition of critical state satisfies Noll’s axioms of determinism and local action in constitutive theory. These axioms define the stress at a point in a body at a certain instant as a function of that point’s kinematic history [31,32], without presupposing any possible future event. Under these axioms, changes in density and stress can occur at critical state. The proposed definition of critical state permits migration from one critical state to another, which occurs in convergent and divergent steady flows [20].

The present theory builds on Coulomb’s contributions to strength, without exclusion or revision. Terzaghi interpreted cohesion and internal friction as purely empirical measures in his theory of over-compacted soil strength (see Appendix A). His theory does not distinguish the orientations of the rupture and slip planes and does not treat normal compaction strength as an independent state parameter. The practical effect is that its domain of application is limited to soil bodies throughout which the state of compaction is uniform. To broaden that domain to include bodies throughout which the state of compaction varies, we need to distinguish the orientations of the rupture and slip planes and relate rupture strength to normal compaction strength (Equation (A20)).

Treating the difference in orientations of the rupture and slip planes as a material property is not new. Jenike and Shield modeled this difference in their paper [9]. Equations (41), (56) and (57) confirm that coincidence of orientations dismisses all interlocking strength and discards any influence of normal compaction. Jenike and Shield also derived the range of specific volume increments possible at critical state for boundary conditions encountered in hoppers. Moreover, they noted that oscillations are commonplace in steady flow within steep hoppers [9]. Jenike’s clarification of his definition of critical state accommodates the changes in stress that occur under such conditions [20].

The present theory is not only fully compatible with the Jenike research program, but broadens the scope of its domain of application. Jenike and Shield assumed that the intermediate principal stress is equal to the average of major and minor principal stresses and that normal compaction strength and critical mean-normal stress are identical:

Γ(ω) = N,(52)

α_0_’ = −1. (53)

Their phase transition function follows directly from Equation (20):

a(ω) = 1.(54)

The expression for critical-compaction strength follows directly from Equations (31) and (54):

σ_cs_’ = g(ω)·[α’ − c_s_·cos φ·(2ω − 1)/3]. (55)

The program’s mobilization condition for critical state is the Mohr-Coulomb envelope. The locus of critical states is a unique surface in ν-p’-τ-ω space, which projects to a unique critical-compaction line in ν-p’ subspace. The expressions for rupture strength at any intermediate principal stress follow directly from Equations (44), (46) and (54)

τ_r_ = c_r_ + σ_nr_’·tan ψ + χ·g(ω)·(α’ − α_0_’), (56)

τ_r_ = [c_s_·cos φ/cos ψ + σ’·tan φ + χ·(σ_cs_’ − σ’)]·cos^2^ ψ  for p_f_’ ≤ p’ ≤ p_cs_’. (57)

The first term on the right-hand side of Equation (56) is the virtual cohesion along the rupture plane, the second term is the internal friction on the rupture plane, and the third term is the normal compaction contribution. The first term within the brackets of Equation (57) is the virtual cohesion along the slip plane, the second term is the internal friction on the slip plane, and the third term is the interlocking strength. The factor cos^2^ ψ adjusts for the change from σ_nr_’ to σ’.

The present theory informs the Cambridge research program by analogy. The program assumes a coherence threshold of

α_0_’ = 0. (58)

The program measures the critical-compaction properties in ν-ln p’ space for an apparent flexibility of

f(p’, ν) = λ/p’  for 0 < p’ ≤ α’.(59)

The reference specific volume in continuous-flow is constant-valued:

Γ(ω) = Γ,(60)

where Γ is one of the program’s fundamental constants. Its phase-transition function follows from Equation (17):

a(ω) = e^−[N−Γ]/(λ−κ)^. (61)

Its critical-compaction surface follows from Equations (16), (58) and (61):

p_cs_’ = e^−[N−Γ]/(λ−κ)^·α’. (62)

The mobilization condition on the slip plane is the cohesionless version of the Drucker–Prager yield condition [19]:

q = M·p’,(63)

where q is the shear stress invariant and M is the coefficient of internal friction (another fundamental constant). The program’s critical-state surface spans ν-p’-q-ω space and projects as a unique line in ν-p’-q subspace. This line’s uniqueness follows directly from Equation (60).

Any change in specific volume due to migration from one critical state to another along this critical-state surface can be related to the change in the mean-normal effective stress. The change in specific volume follows from Equations (4) and (59):

dν_cs_ = − λ·dp_cs_’/p_cs_’. (64)

The expression for rupture strength pursuant to Schofield’s conjecture [17] follows from adopting the original Drucker–Prager envelope as the mobilization condition on the rupture plane:

q = M·p’ + Χ·{e^−[N−Γ]/(λ−κ)^·α’ − p’}  for p_f_’ ≤ p’ ≤ p_cs_’, (65)

where X is the coefficient of interlocking strength:

Χ ≡ M − R.(66)

R is the coefficient of internal friction along the rupture plane. It is the slope in p’-q subspace of the projection of the rupture surface. Χ is a material constant. The first term on the right-hand side of Equation (65) is Schofield’s internal friction (that is, the internal friction along the slip plane); the second term is the interlocking strength.

Adding cohesion C to the mobilization condition satisfies Dean’s request [18]. This addition does not affect either the internal-friction term or the interlocking strength term and yields

q = C + M·p’ + Χ·{e^−[N−Γ]/(λ−κ)^·α’ − p’}   for p_f_’ ≤ p’ ≤ p_cs_’. (67)

The equivalent equation for rupture strength expressed in terms of the coefficient of internal friction along the rupture plane is

q = C + R·p’ + Χ·e^−[N−Γ]/(λ−κ)^·α’  for p_f_’ ≤ p’ ≤ p_cs_’. (68)

We can extend this solution to include any observed dependency on shearing mode by redefining the three material constants (M, R, and Γ) as material functions of the shearing mode.

The development of rupture strength here (Equations (45), (46), (56), (57), (67) and (68)) incorporates normal compaction strength without reference to kinematics. The definition of interlocking strength associates the vanishing of interlocking with a perfect balance of mean-normal effective stress and critical-compaction strength. Equations (32) and (64) relate changes in specific volume to changes in mean-normal effective stress and shearing mode without reference to shearing strain. Shearing strain and volume change are uncoupled at critical state. In other words, the present theory is open to the modeling of viscous effects through the mobilization envelope.

### 3.2. Dilation

When a granular material ruptures, it dilates. Terzaghi’s theory excludes dilation from the outset by assuming perfect plasticity. The present theory does not assume perfect plasticity. From its standpoint, dilation and strength are distinct features. Interlocking is the strength due to over-compaction. Taylor’s interlocking, on the other hand, is a kinematic measure of dilation that couples the change in volumetric strain to the change in shearing strain. The present theory does not assume any kinematic coupling.

Taylor’s interlocking is fundamental to theories that explain dilation [7,8,10,15,22,23,24,25,26]. Rowe [10] distinguished the discrete planes of inter-particle sliding from an imaginary plane of particle interlocking and treated the difference in their orientations as a solution parameter. He remarked that, if the Mohr-Coulomb criterion of failure were to be applied to regular packings, it would be necessary to replace the discrete planes by an equivalent single plane at angle 45-φ/2 to the major principal axis. In his analysis, he predicted the coincidence of these discrete planes with the imaginary plane at ultimate failure based on minimum internal work. It is important to note that any coincidence of the discrete planes with the imaginary plane at critical state does not imply the coincidence of the rupture and slip planes at that state. The present theory does not assume that the Mohr-Coulomb theory of strength requires coincidence of the orientations of the rupture and slip planes. Indeed, the present theory, by modeling the difference in the orientations of these planes as a material constant, offers the following solution to the Terzaghi-Schofield disagreement:

- Define interlocking in terms of the difference between the critical mean-normal effective stress and the mean-normal effective stress in place of Taylor’s ratio.
- Distinguish any virtual cohesion from the contributions due to over-compaction.

Note that this solution does not preclude using Taylor’s ratio to explain the dilation that occurs as the material ruptures.

The normal compaction strength in the present theory is related to the state parameter ξ recently introduced by Collins and his collaborators [15]. Their parameter measures the fraction of applied plastic volumetric work released during dilation. Their theory assumes critical-compaction constants measured in ln ν-ln p’ space:

ξ = (Γ/N)^−^^1/(β^^−ζ^^)^·α’/p’. (69)

The interchangeable equations for rupture strength may be expressed in terms of ξ:

q = C + R·p’ + Χ·ξ·p’  for p_f_’ ≤ p’ ≤ p_cs_’, (70)

q = C + M·p’ + Χ·(ξ − 1)·p’  for p_f_’ ≤ p’ ≤ p_cs_’. (71)

The terms on the right-hand side of Equation (70) are, respectively, the virtual cohesion, the internal friction on the rupture plane and the normal compaction contribution. The terms on the right-hand side of Equation (71) are, respectively, the virtual cohesion, the internal friction on the slip plane and the interlocking strength. Their theory addresses cohesionless soils:

q = R·p’ + X·ξ·p’  for p_f_’ ≤ p’ ≤ p_cs_’, (72)

q = M·p’ + Χ·(ξ − 1)·p’  for p_f_’ ≤ p’ ≤ p_cs_’. (73)

The present theory’s fracture bound provides an upper bound on the value of the Collins parameter. For example, for cohesionless soil under triaxial compression,

1 ≤ ξ ≤ (3 − R)/Χ  for ω = 0.(74)

If M ~ 1 and R ~ 0.5, the range for dilatant behavior is 1 ≤ ξ ≤ 5.

The kinematic theories that explain dilation couple volumetric change to shearing strain and assume that volumetric straining at critical state is purely reversible. The present theory does not couple volumetric change to shearing strain and predicts specific volume changes at critical state that consist of both reversible and irreversible components. The structural component of any specific volume change at critical state for properties defined in ln ν-ln p’ space is given by

dν_cs|s_ = − (β − ζ)·ν·dp_cs_’/(p_cs_’ − α_0_’). (75)

If shearing mode remains constant, α’/p’ remains constant and hence so does ξ.

The critical state defined in the present theory differs from the Reynolds–Taylor state defined by Collins and his collaborators [22]. The present theory treats critical state as a singularity with respect to coupling of volume change and shearing strain.

### 3.3. Comparison

Table 1 below compares the present theory with those of Jenike and Shield, Schofield, and Collins and his collaborators. The Jenike and Shield theory is a special case of the present theory.

## 4. Materials and Methods

These results can be reproduced without additional materials or special methods.

## 5. Conclusions

The present paper has developed a theory of rupture strength for granular materials. This theory retains the original features of the Mohr-Coulomb theory of strength. The present theory has incorporated the particulate nature of a granular material by distinguishing between rupture and slip planes of failure. This theory has introduced normal compaction strength as a new state parameter. It has augmented cohesion and internal friction with a normal compaction contribution to shear strength. Rupture strength is expressed in two interchangeable forms: (a) as the sum of cohesion along the rupture plane, internal friction along the rupture plane, and a normal compaction contribution; and (b) as the sum of cohesion along the slip plane, internal friction along the slip plane, and an interlocking strength. The locus of rupture states describes a surface that is bounded by fracture and critical-state surfaces in state space.

This paper, in developing its theory of rupture strength, has elaborated the concept of critical state, which is well-established in physics and chemistry, to include granular materials. This provides a common platform for identifying a critical state of any material, granular or not. The critical state surface of a material is the locus of its multi-phase states at which the densities of the phases coincide. This surface is the intersection of the critical-compaction and mobilization surfaces for the material. The equations that define these surfaces represent the necessary and sufficient conditions for a critical state.

The material’s critical-compaction surface partitions state space into over-compacted and lightly compacted domains without regard to its shear strength. The material’s mobilization surface identifies the states that mobilize its internal friction without regard to its state of compaction.

This paper has reconciled the classical works by Roscoe and his colleagues on critical state and the works by Jenike. It has offered a solution to the Terzaghi-Schofield disagreement and has suggested enhancements to the Cambridge and Jenike research programs. It has distinguished the interlocking that contributes to a material’s shear strength and Taylor’s interlocking that measures the material’s dilation at rupture. Finally, this paper has identified the relation between normal compaction strength and the state parameter that Collins and his colleagues [15] recently introduced into their thermomechanical theory of dilation.

## Figures and Tables

**Table 1:** Comparison of theories of rupture strength for a granular material.

Feature	Present Theory	Jenike–Shield	Schofield	Collins et al.
Non-teleological	Yes	Yes	No	No
Reference stress	α’	α’ = p_cs_’ = σ_cs_’	p_cs_’	p_cs_’
Shearing mode	Yes	Yes	No	No
Cohesion	Yes	Yes	No	No
Range of σ_2_’	σ _3_’ ≤ σ_2_’ ≤ σ_1_’	σ_2_’ = (σ_1_’ + σ_3_’)/2	σ_2_’ = σ_3_’	σ_2_’ = σ_3_’
Locus of critical states is a	Surface	Surface	Line	Line
Strain rates at critical state	Uncoupled	Uncoupled	Coupled	Coupled
Allows rate dependence	Yes	Yes	No	No
Elasto-plastic, rigid-plastic	Elasto-plastic	Rigid-plastic	Elasto-plastic	Elasto-plastic
Parallel flow	Yes	Yes	Yes	Yes
Non-parallel flow	Yes	Yes	No	No
New state parameter	α’	α’	?	ξ

## Glossary/Nomenclature/Abbreviation

**Parameters**	
α’	normal compaction strength, maximum mean-normal effective stress
α_0_’	minimum mean-normal effective stress for well-defined flexibilities
ν	specific volume
ν_cs_	specific volume at critical compaction
σ_1_’	major principal effective stress
σ_2_’	intermediate principal effective stress
σ_3_’	minor principal effective stress
σ’	average of the major and minor principal effective stresses
σ_f_’	σ’ at the intersection of the fracture and rupture surfaces
σ_nr_’	normal effective stress component on the rupture plane
σ_ns_’	normal effective stress component on the slip plane
σ_cs_’	average of major and minor principal effective stresses at critical state
τ_r_	shear strength component along the rupture plane
τ_s_	shear strength component along the slip plane
ω	shearing mode
p’	mean-normal effective stress
p_0_’	reference mean-normal effective stress
p_f_’	p’ at the intersection of the fracture and rupture surfaces
p_cs_’	critical-compaction strength—p’ at critical state
r	radius of Mohr’s circle of stress
**Material Properties**	
β	index of ln-ln normal compaction line in ν-p’ space
Γ(ω)	specific volume at p_0_’ on the continuous-flow line for ω
Γ	specific volume at p_0_’ on the continuous-flow line—a material constant
ξ	index of ln-ln swelling line in ν-p’ space
κ	index of semi-ln swelling line in ν-p’ space
λ	index of semi-ln normal compaction line in ν-p’ space
φ	angle of internal friction on the slip plane
ψ	angle of internal friction on the rupture plane
χ	coefficient of interlocking strength
a(ω)	phase-transition function
c_r_	virtual cohesion along the rupture plane
c_s_	virtual cohesion along the slip plane
f(p’,ν)	apparent flexibility—slope of the normal compaction line
f_c_(p’,ν)	centric flexibility—slope of the swelling line
g(ω)	intermediate principal effective stress factor
m	coefficient of compaction influence
M	coefficient of internal friction on the slip plane—Cambridge research program
N	specific volume at p_0_’ on the normal compaction line
P	coefficient of interlocking—Cambridge research program
R	coefficient of internal friction on the rupture plane—Cambridge research program
Χ	coefficient of interlocking strength—Cambridge research program
**Terzaghi’s Implementation—equivalent terms are shown in parentheses**	
α	normal compaction strength
α_0_	coherence threshold for well-defined flexibilities
σ	normal stress under normally compacted conditions (σ_ns_)
σ’	normal stress under over-compacted conditions (σ_nr_)
σ_1_	major principal stress
σ_3_	minor principal stress
σ_cs_	average of major and minor principal stresses at critical state
τ	shear strength under normally compacted conditions (τ_s_)
τ’	shear strength under over-compacted conditions (τ_r_)
φ	angle of internal friction under normally compacted conditions (φ)
φ’	angle of internal friction under over-compacted conditions (‘true’) (ψ)
φ_f_	angle of internal friction in Terzaghi’s Equation (5.3) [5]
c	cohesion under normally compacted conditions (c_s_)
c’	effective cohesion under over-compacted conditions (‘true’) (c_r_ + m·(α − α_0_))
c_0_’	virtual cohesion under over-compacted conditions (c_r_)
N	gradient of the effective cohesion relation in Terzaghi’s Equation (5.3) [5]
s	shear strength in Terzaghi’s implementation

## Appendix A. Terzaghi’s Theory of Strength

Terzaghi, in his text entitled Theoretical Soil Mechanics [5], introduces his theory of strength assuming that “the relation between the normal stress σ on every section through a mass of cohesive soil and the corresponding resistance s per unit of area can be represented by an empirical equation”. He identifies this equation as Coulomb’s equation:

s = c + σ·tan φ. (A1)

He uses total stress terms throughout and assumes “that plastic flow, which involves continuous deformation under constant stress, has no influence on the values of cohesion or angle of internal friction”. His notation is used here.

Terzaghi defines the shear strength of a soil element under normally compacted conditions by Equation (A1) and the shear strength of the same soil element under over-compacted conditions as

s = c’ + σ·tan φ’, (A2)

where ’ identifies the over-compacted coefficients; and c, φ, c’ and φ’ are empirical constants: “c’ is considerably higher than c and φ’ is considerably smaller than φ”. He attributes his usage of the term cohesion to “historical reasons only. It is used as an abbreviation of the term apparent cohesion. In contrast to the apparent cohesion, the true cohesion represents that part of the shearing resistance of a soil which is a function only of the water content. It includes not only c in Coulomb’s equation but also an appreciable part of σ tan φ”. He decomposes the added strength under over-compacted conditions into “two parts with different physical causes”. The first part is the friction produced by the normal stress σ and the second part is the increase of the cohesion due to the reduction of the water content which occurred while the pressure on the specimen was increased from zero to σ”. His Equation 5.3 [5] states

s = c + σ·tan φ = c + [(σ_1_ + σ_3_)/2]·N + σ·tan φ_f_. (A3)

N is an empirical factor and φ_f_ varies with the orientation of a section through the element. He notes that determination of N and φ_f_ requires “elaborate supplementary investigations which belong to the realm of soil physics”. The second term on the right-hand side is the part of “σ tan φ” borrowed to augment c. He defines true cohesion as “equal not to c but to”

c’ = c + [(σ_1_ + σ_3_)/2]·N. (A4)

The second term on the right-hand side represents the increase in strength due to compaction.

Consider distinguishing the rupture plane from the slip plane for the same state. The shear strength under normally compacted conditions is given by (cf. Equation (A1))

τ = c + σ_ns_·tan φ. (A5)

The shear strength under over-compacted conditions is given by (cf. Equation (A2))

τ’ = c’ + σ_nr_·tan φ’. (A6)

These shear strength components expressed in terms of σ are given by

τ = c·cos^2^ φ + σ·sin φ·cos φ, (A7)

τ’ = c’·cos^2^ φ’ + σ·sin φ’·cos φ’. (A8)

Mohr’s circle of stress relates these components on the slip and rupture planes for the same state

τ’ = τ·cos φ’/cos φ. (A9)

Equation (A4) may be rewritten as

c’ = c + N·σ. (A10)

Substituting Equations (A7), (A8) and (A10) into Equation (A11) yields

c·cos φ = (N·cos φ’ − sin φ + sin φ’)·σ + c·cos φ’. (A11)

Since c, φ and φ’ are independent of σ, the parentheses-enclosed terms must vanish. Hence,

N = (sin φ − sin φ’)/cos φ’. (A12)

Substituting Equation (A12) into Equation (A11) gives

φ’ = φ. (A13)

Equation (A4) effectively merges the rupture and slip planes.

Terzaghi, in explaining his Equation 5.3 [5], states that “the most conspicuous permanent change produced by pre-consolidation consists in an increase of the density of the material and a corresponding reduction in its water content”. Let us implement this hypothesis by replacing his Equation (A4) with an equation that is linearly related to normal compaction strength, α:

c’ = c_0_’ + m·(α − α_0_). (A14)

c_0_’ and α_0_ are material constants. The state that supports both slip and rupture is the transition state between lightly compacted and over-compacted conditions identified by

σ_cs_ = g(ω)·{a(ω)·α + [1 − a(ω)]·α_0_ − c·cos φ·(2ω − 1)/3}. (A15)

Substituting Equations (A7), (A8), (A14) and (A15) into Equation (A9) yields

        c·cos φ [1 − g(ω)·(sin φ − sin φ’)·(2ω − 1)/3]             = c_0_’·cos φ’ + [m·cos φ’ − a(ω)·g(ω)·(sin φ − sin φ’)]·α        − [m·cos φ’ − a(ω)·g(ω)·(sin φ − sin φ’)]·α_0_ − g(ω)·(sin φ − sin φ’)·α_0_. (A16)

Since c, φ, c_0_’ and φ’ are independent of normal compaction strength, the brackets-enclosed second term on the right-hand side must vanish. Hence,

m = a(ω)·g(ω)·(sin φ − sin φ’)/cos φ’. (A17)

Substituting Equation (A17) into Equation (A16) relates the rupture constant, c_0_’, to normally compacted cohesion, c,

             c_0_’·cos φ’ = c·cos φ [1 − g(ω)·(sin φ − sin φ’)·(2ω − 1)/3] + g(ω)·(sin φ − sin φ’)·α_0_. (A18)

Substituting Equation (A17) into Equation (A14) and the result into Equation (A8) gives

τ’ = c_0_’·cos^2^ φ’ + σ·sin φ’·cos φ’         + a(ω)·g(ω)·(sin φ − sin φ’)·(α − α_0_)·cos φ’. (A19)

Rearranging the last two terms on the right-hand side and substituting Equations (A15) and (A18) gives

τ’ = [c + σ·tan φ + (σ_cs_ − σ)·(sin φ − sin φ’)/cos φ] cos φ·cos φ’. (A20)

The third term within the brackets is the interlocking strength term missing in Terzaghi’s theory of strength.

## References

[1] Yu M. (2002). Advances in strength theories for materials under complex stress state in the 20th century. Appl. Mech. Rev..

[2] Coulomb C.A. (1776). Essai sur une Application des Règles de Maximis et Minimis à Quelques Problèmes de Statique, Relatifs à l’Architecture.

[3] Heyman J. (1972). Coulomb’s Memoir on Statics: An Essay in the History of Civil Engineering.

[4] Mohr O. (1900). Welche Umstande bedingen die Elastizitatsgrenze und den Bruch eines Materials. Z. Ver. Deutsch. Ing. Band.

[5] Terzaghi K. (1943). Stress Conditions for Failure in Soils. Theoretical Soil Mechanics.

[6] Reynolds O. (1885). On the dilatancy of media composed of rigid particles in contact. Philos. Mag..

[7] Taylor D.W. (1948). The Fundamentals of Soil Mechanics.

[8] Roscoe K.H., Schofield A.N., Wroth C.P. (1958). On the Yielding of Soils. Geotechnique.

[9] Jenike A.W., Shield R.T. (1959). On the plastic flow of Coulomb solids beyond original failure. ASME J. Appl. Mech..

[10] Rowe P.W. (1962). The stress-dilatancy relation for static equilibrium of an assembly of particles in contact. Proc. R. Soc. Lond. A.

[11] Schofield A.N., Wroth C.P. (1968). Critical State Soil Mechanics.

[12] Parry R.H.G. (1960). Triaxial compression and extension tests on remoulded saturated clay. Geotechnique.

[13] Jenike A.W. (1988). Personal Communication with the Author.

[14] Prager W. (1949). Recent Developments in the Mathematical Theory of Plasticity. J. Appl. Phys..

[15] Collins I.F., Muhunthan B., Qu B. (2010). Thermomechanical state parameter models for sands. Geotechnique.

[16] Schofield A.N. (1999). A note on Taylor’s interlocking and Terzaghi’s “true cohesion” error. Geotechnical News.

[17] Schofield A.N. (2006). Interlocking, and peak and design strengths. Geotechnique.

[18] Burland J.B., Dean E.T.R., Gudehus G., Muhunthan B., Collins I. F. (2008). Discussion: Interlocking, and peak and design strengths. Geotechnique.

[19] Drucker D.C., Prager W. (1952). Soil mechanics and plastic analysis for limit design. Q. Appl. Math..

[20] Jenike A.W. (1987). A theory of flow of particulate solids in converging and diverging channels based on a conical yield function. Powder Technol..

[21] Yin Z., Chang C.S. (2009). Non-uniqueness of critical state line in compression and extension conditions. Int. J. Numer. Anal. Meth. Geomech..

[22] Kanatani K.-I. Mechanical foundation of the plastic deformation of granular materials. Proceedings of the IUTAM Conference on Deformation and Failure of Granular materials.

[23] Goddard J.D., Bashir Y.M., DeKee D., Kaloni P.N. (1990). On Reynolds dilatancy. Recent Developments in Structured Continua.

[24] Houlsby G.T., Kolymbas D. (1993). Interpretation of dilation as a kinematic constraint. Modern Approaches to Plasticity.

[25] Dafalias Y.F. (1986). An anisotropic critical state clay plasticity model. Mech. Res. Commun..

[26] Collins I.F., Muhunthan B., Tai A.T.T., Pender M.J. (2007). The concept of a ‘Reynolds-Taylor state’ and the mechanics of sands. Geotechnique.

[27] Wroth C.P., Bassett R.H. (1965). A stress-strain relationship for the shearing behavior of a sand. Geotechnique.

[28] Been K., Jefferies M.G. (1985). A state parameter for sands. Geotechnique.

[29] Poole C.P. (2004). Encyclopedia of Condensed Matter Physics.

[30] Schofield A.N. (2005). Disturbed Soil Properties and Geotechnical Design.

[31] Noll W. (1958). A mathematical theory of the mechanical behavior of continuous media. Arch. Ration. Mech. Anal..

[32] Truesdell C. (1977). Constitutive Relations. A First Course in Rational Continuum Mechanics.

